# Impact of Common Cleaning Protocols on the Light Transmittance of Polyethylene Terephthalate Glycol (PET-G) Clear Aligners: An In Vitro Study

**DOI:** 10.7759/cureus.110546

**Published:** 2026-06-09

**Authors:** Armen Zohrab Yeretsian, Ahmad S Burhan, Mohammad Y Hajeer, Samer T Jaber, Fehmieh R Nawaya, Alaa Oudah Ali Almusawi

**Affiliations:** 1 Department of Orthodontics, Faculty of Dentistry, University of Damascus, Damascus, SYR; 2 Department of Orthodontics, Faculty of Dentistry, Al-Wataniya Private University, Hama, SYR; 3 Department of Pediatric Dentistry, Faculty of Dentistry, Syrian Private University, Damascus, SYR; 4 Department of Orthodontics, Faculty of Dentistry, University of Al-Knooz, Basrah, IRQ

**Keywords:** cleaning protocols, light transmittance, optical properties, pet-g clear aligners, spectrophotometry

## Abstract

Background

The optical clarity of clear aligners is essential for esthetics, and routine cleaning protocols may alter their light transmittance. This study evaluated the effect of commonly used cleaning methods on the light transmittance of polyethylene terephthalate glycol (PET-G) clear aligners in vitro.

Materials and methods

Forty PET-G specimens (10 × 10 mm) were divided into four groups (n = 10): Control, Listerine® rinse (Johnson & Johnson, Neuss, Germany), Corega® effervescent tablets (Stafford-Miller, Dungarvan, Ireland), and toothbrush with Colgate® toothpaste (Colgate-Palmolive, Guangzhou, China). The specimens were subjected to the assigned cleaning protocols once daily for 14 days. Light transmittance was measured using a UV-Vis-NIR (Ultraviolet-Visible-Near Infrared) spectrophotometer. Data were analyzed using the Shapiro-Wilk test, Levene’s test, Welch’s ANOVA, and Games-Howell post hoc comparisons at α = 0.05. Measurement reproducibility was assessed using repeated measurements and the intraclass correlation coefficient (ICC).

Results

Measurement reproducibility was excellent, with no significant systematic difference between repeated measurements (P = 0.515) and an ICC of 0.993. The highest mean light transmittance was observed in the Listerine group (87% ± 2%), followed by the control and Corega groups (85% ± 3% each), whereas the toothbrush group showed the lowest value (64% ± 6%). Levene’s test indicated unequal variances (P = 0.001), and Welch’s ANOVA showed a significant difference among groups (P < 0.001). Games-Howell post hoc analysis revealed that the toothbrush group had significantly lower transmittance than all other groups (P < 0.001), while no significant differences were found between the control, Corega, and Listerine groups.

Conclusion

Toothbrush-and-toothpaste cleaning markedly reduced the light transmittance of PET-G clear aligners, whereas chemical cleaning methods preserved optical properties. Non-abrasive cleaning approaches may be preferable for maintaining aligner transparency.

## Introduction

The increasing demand for "invisible" orthodontic treatments among adults has led to a significant growth in the clear aligner market [1]. These aligners are favored for their low aesthetic impact, effectiveness in guiding teeth, and removability, which simplifies oral hygiene maintenance [1]. Clear aligners are typically made from resin polymers such as polyethylene terephthalate glycol (PET-G), polypropylene (PP), polycarbonate (PC), and thermoplastic polyurethanes (TPU) [1,2]. Among these materials, PET-G-based materials often exhibit the highest transparency in the visible light spectrum [3]. Maintaining this optical clarity is essential, as the esthetic benefit of aligners depends on high light transmittance, ensuring they remain inconspicuous on the teeth. Clinicians and patients must see through the aligner to check fit and hygiene; conversely, any loss of transparency (yellowing or cloudiness) undermines treatment acceptance.

Clear aligners are subject to changes within the oral environment, including warmth, humidity, mastication forces, and prolonged exposure to salivary enzymes [1]. The manufacturing process itself, known as thermoforming, can also alter the mechanical, optical, chemical, and morphological properties of the aligner materials [4]. Studies of in vivo-aged aligners show that even new aligners have micro‐irregularities (tiny scratches and grooves) that promote biofilm adhesion. After two weeks of use, PET-G aligners show signs of microcracking, abrasion marks, delamination, calcified biofilm deposits, and significant loss of clarity [5]. Pigmentation, thermal stress, and other environmental factors cause discoloration, roughening, and microstructural changes in thermoplastics [6]. Therefore, while starting with excellent clarity, intraoral wear and poor cleanliness might severely compromise PET-G transparency.

Maintaining clear aligners is important for oral health, as unwashed aligners can promote bacterial biofilms, leading to bad breath, oral infections, and plaque buildup [5]. Various methods for keeping appliance surfaces clean are described: mechanical brushing with or without toothpaste, chemical disinfectant soaks, effervescent cleaning tabs, ultrasonic bath treatment, and even rinsing with solutions containing alcohol or soap [5,7]. For instance, according to systematic reviews, mechanical cleaning (brushing/vibration), chemical agents (chlorhexidine, anionic detergents, peroxide tabs), and their combinations have been studied [5]. The mechanisms of action of these protocols vary, and each may affect the polymer surface, potentially influencing light transmission.

There have been relatively few studies evaluating the light transmittance of aligners. For example, Lombardo et al. compared the light transmittance of three commercial aligner brands using spectrophotometry, both before and after artificial aging [1]. It was concluded that although some reduction in light transmittance occurred over 28 days of aging (not statistically significant), baseline transparency varied: one material (F22) was significantly more transparent than others. A recently published paper on the aging of nine different materials also reported a clear deterioration in the optical properties of aligners [8]. Additionally, some previous studies evaluated changes in color due to specific external factors. For example, exposure to cigarette smoke reduced Invisalign light transmittance from ~68% to ~60% [9]. In these studies, measurements were performed using UV-Vis (Ultraviolet-Visible) spectrophotometry or colorimetry (e.g., CIELab), often on flat specimens or sectioned aligner samples [1,6]. Most protocols assess light transmittance in the visible range (often ~500 nm) and calculate the total visible light transmission [6]. Such protocols allow for the detection of subtle changes in material clarity.

These previous studies have shown that stains and aging can compromise aligner transparency and that light transmittance is a sensitive indicator of such material changes. However, the existing evidence has several limitations. Many studies have used mixed-brand or unknown polymers (not always pure PET-G) [1,9]. In addition, variations in sample preparation, such as the use of full aligners versus flat specimens, and the focus on single cleaning or aging factors, limit comparability. Consequently, the effect of commonly used cleaning protocols on the light transmittance of PET-G aligners has not been systematically investigated.

In summary, although the invisibility of clear aligners depends on the optical clarity of PET-G, both environmental factors and cleaning procedures may influence their surface characteristics and transparency [5,6]. While there is growing evidence that aging and staining reduce aligner transparency, the effects of routine cleaning protocols on the optical properties of PET-G aligners remain insufficiently understood. This gap is clinically important, as reduced light transmittance may adversely affect both aesthetics and material integrity. Accordingly, this study aimed to evaluate the effect of commonly used cleaning protocols on the light transmittance of PET-G clear aligners in vitro.

## Materials and methods

Study design and settings

This study was designed as a controlled in vitro experimental investigation conducted at the Department of Orthodontics, Faculty of Dentistry, Damascus University, Damascus, Syria. Its primary aim was to evaluate and compare the effects of three commonly used home-care cleaning protocols (Listerine® mouth rinse (Johnson & Johnson, Neuss, Germany), Corega® effervescent tablet immersion (Stafford-Miller, Dungarvan, Ireland), and manual toothbrushing using Colgate® toothpaste (Colgate-Palmolive, Guangzhou, China)) on the light transmittance of PET-G clear aligner specimens (Clear Smile, Damascus, Syria). The study was funded by Damascus University (Funding ID: 501100020595).

Estimation of the sample size

Sample size estimation was performed a priori using G*Power software (version 3.1.9; Heinrich Heine University, Düsseldorf, Germany). A one-way ANOVA test was applied based on the following assumptions: a significance level (α) of 0.05 and a statistical power (1 - β) of 90%. The effect size (f = 0.66) was derived from previously published data by Alweneen et al. [10]. Based on these parameters, a total sample size of 40 specimens was determined to be sufficient for the primary outcome measure. The specimens were then equally allocated into four groups, with 10 samples assigned to each group.

Sample preparation

The study sample consisted of 40 specimens from clear aligners, 0.8 mm thick, fabricated from PET-G (Clear Smile, Damascus, Syria) using the thermoforming pressure technique, according to the manufacturer's instructions (Ministar S®, Scheu-Dental GmbH, Iserlohn, Germany). A single gypsum model was 3D scanned to produce a resin model, onto which a transparent aligner sheet was thermoformed. The molded section was trimmed and separated, then cut into flat rectangular strips measuring 10 × 10 mm for testing, with four specimens obtained from each thermoformed sheet.

The specimens were equally distributed into four groups, each consisting of 10 samples. The groups were classified on the basis of the cleaning method applied: Group 1 served as the control; Group 2 underwent cleaning with Listerine® solution; Group 3 was treated using Corega® cleansing tablets; and Group 4 was cleaned with a toothbrush and Colgate® toothpaste.

Cleaning protocols

Artificial saliva was prepared according to the formulation described by Greenwood, following the method of Imai et al. [11]. Per 100 g of distilled water, the solution contained: potassium chloride (KCl) 0.24 g, calcium phosphate (Ca₃(PO₄)₂) 0.06 g, dipotassium hydrogen phosphate (K₂HPO₄) 0.14 g, potassium sulfate (K₂SO₄) 0.09 g, trisodium phosphate (Na₃PO₄) 0.08 g, and albumin 0.5 g. This composition was used to simulate oral conditions during specimen conditioning and testing. The salts were sequentially dissolved in distilled water with a magnetic stirrer, and albumin was then added at 37°C to ensure complete dissolution. All specimens were initially immersed in artificial saliva within plastic containers and incubated at 37°C for 24 hours using a laboratory incubator. Following this initial conditioning period, the specimens were rinsed with distilled water for one minute to remove residual saliva before applying the cleaning protocols. The cleaning procedures described below were performed once daily for 14 consecutive days, corresponding to the typical clinical service duration of a single aligner.

Group 1 - Control (No Cleaning Intervention)

The specimens in the control group did not undergo any active cleaning procedure. They were handled and rinsed in the same manner as the experimental groups (distilled water rinse and air-drying), but were otherwise left untreated and stored in artificial saliva between cycles.

Group 2 - Listerine® Mouthwash

**Figure 1:**
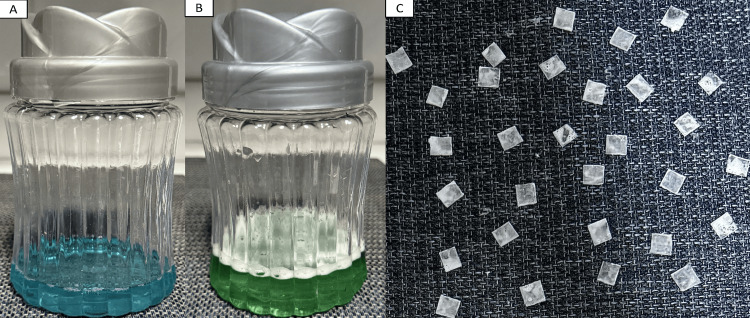
Representative images of the three cleaning protocols applied to polyethylene terephthalate glycol (PET-G) clear aligner specimens. A: Immersion in Listerine® mouthwash. B: Immersion in Corega® effervescent solution. C: Manual toothbrushing with Colgate® toothpaste.

The specimens were immersed in Listerine mouthwash for 15 minutes (Figure 1A), rinsed with distilled water for one minute, and then air-dried. After drying, the specimens were returned to artificial saliva and incubated at 37°C until the next daily cycle.

Group 3 - Corega® Cleansing Tablets

Corega tablets were dissolved in warm water according to the manufacturer’s instructions. The specimens were immersed in the prepared solution for three minutes (Figure 1B), rinsed with distilled water for one minute, air-dried, and then returned to artificial saliva and incubated at 37°C.

Group 4 - Toothbrushing With Colgate® Toothpaste

The specimens in this group were manually cleaned using a soft-bristle Colgate toothbrush and Colgate toothpaste (Figure 1C). A pea-sized amount of toothpaste was prepared as a slurry and applied to the brush head prior to each cleaning cycle. Each specimen was brushed for 30 seconds per surface (two surfaces per specimen; 60 seconds total) using circular motion, with the brush head held approximately perpendicular (≈90°) to the specimen surface. Brushing force was applied manually based on the weight of the toothbrush. To minimize operator-related variability, all procedures were performed by the same operator, the brushing time was standardized using a stopwatch, and the toothpaste slurry was refreshed between specimens. After brushing, specimens were rinsed with distilled water for one minute, air-dried, and subsequently returned to artificial saliva and incubated at 37°C until the next cycle.

Outcome measures: light transmittance test

Light transmittance was measured on flat, square specimens (10 mm × 10 mm) following completion of the assigned cleaning protocols for both the control and experimental groups. Measurements were performed using an Agilent Cary 5000 UV-Vis-NIR (Ultraviolet-Visible-Near Infrared) spectrophotometer equipped with a Diffuse Reflectance Accessory (DRA-2500) integrating sphere. Spectral scanning was conducted over the wavelength range of 350-800 nm, covering the visible and near-infrared regions, and percentage light transmittance (%T) was recorded as a function of wavelength.

Prior to measurement, the instrument was calibrated by recording a baseline reference without a specimen (100% transmittance). A single spectral scan was performed for each specimen due to standardized, homogeneous sample preparation. All measurements were carried out under controlled laboratory conditions, and data acquisition and analysis were performed using Cary WinUV software (Agilent Technologies, Santa Clara, CA). Transmittance values were extracted at representative wavelengths (e.g., 400, 550, and 700 nm), in addition to generating full spectral transmittance curves for each group.

Blinding

Blinding during the cleaning interventions was not feasible due to the inherent characteristics of the protocols used. However, blinding was implemented during the outcome assessment. All the specimens were coded prior to light transmittance analysis to conceal group allocation. Light transmittance measurements were performed using an instrument-based protocol by an investigator who was not involved in specimen cleaning or coding, thereby maintaining allocation concealment. The specimens were analyzed in randomized order following standardized rinsing and drying procedures to ensure uniform surface conditions before measurement. Although certain cleaning protocols may induce subtle visible surface alterations, investigators had no access to allocation data at the time of testing.

Assessment of measurement reproducibility

An exploratory reproducibility assessment was performed on twenty specimens to evaluate the precision and agreement of the measured variables. All the measurements were repeated after 24 hours under identical laboratory conditions. Systematic (bias) error was assessed using paired-samples t-tests at a 95% confidence level, whereas random error and inter-measurement agreement were quantified using the intraclass correlation coefficient (ICC) (two-way mixed model, absolute agreement).

Statistical analysis

All statistical analyses were performed using IBM® SPSS Statistics version 26.0 (IBM Corp., Armonk, NY). The light transmittance values are presented as the mean ± standard deviation (SD). The normality of the data was assessed using the Shapiro-Wilk test. The assumption of homogeneity of variances was evaluated using Levene’s test and was found to be violated. Therefore, between-group comparisons were performed using Welch’s ANOVA, followed by Games-Howell post hoc pairwise comparisons. The level of significance was set at α = 0.05.

## Results

Assessment of measurement reproducibility

No statistically significant systematic differences were observed between the first and second measurements for light transmittance (P = 0.515), indicating the absence of systematic error. Reliability was excellent, with an ICC of 0.993, confirming the high repeatability and consistency of the measurement method used (Table 1). 

**Table 1:** Assessment of measurement reproducibility for light transmittance (paired t-test and intraclass correlation coefficient). CI: confidence interval; ICC: intraclass correlation coefficient; SD: standard deviation; *: P-value for paired t-test; **: P-value for ICC

Variable	Mean ± SD (Prior to Initiation)	Mean ± SD (After 24 Hours)	Mean Difference	95% CI for Difference (%)	P-value*	ICC	95% CI for ICC	P-value**
Light transmittance	85% ± 2%	85% ± 3%	0.1%	-0.1 to 0.3	0.515	0.99	0.97 to 1.00	< 0.001

Main findings

**Figure 2:**
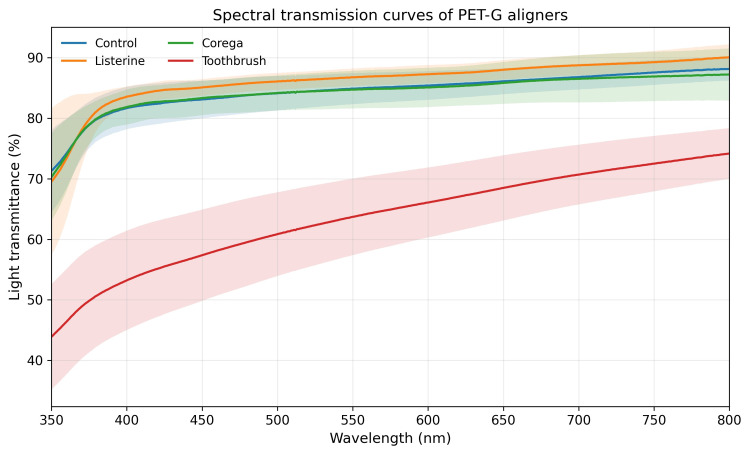
Spectral transmission curves of polyethylene terephthalate glycol (PET-G) clear aligner specimens after 14 days of daily cleaning protocols.

Descriptive statistics and normality tests are presented in Table 2, while the post hoc pairwise comparisons are shown in Table 3. The wavelength-specific light transmittance values (mean ± SD) for all groups across the 350-800 nm range are presented in Table 4. The highest mean light transmittance was recorded in the Listerine group (87% ± 2%), followed by the Control and Corega groups, which exhibited identical mean values (85% ± 3%). In contrast, the Toothbrush group demonstrated a markedly lower mean light transmittance (64% ± 6%), representing the lowest value among all groups. The spectral transmission curves for all groups are displayed in Figure 2.

**Table 2:** Descriptive statistics and results of normality (Shapiro-Wilk) test for light transmittance. SD: standard deviation; *: Shapiro-Wilk test

Groups	Mean ± SD	P-value*
Control	85% ± 3%	0.197
Corega	85% ± 3%	0.092
Listerine	87% ± 2%	0.668
Toothbrush	64% ± 6%	0.982

**Table 3:** Post hoc tests for pairwise comparisons of light transmittance. *Statistically significant difference according to Games-Howell’s post hoc test (p < 0.05).

Groups	Light Transmittance		
	Corega	Control	P-value
Corega vs. Control	85% ± 3%	85% ± 3%	1.00
Corega vs. Toothbrush	85% ± 3%	64% ± 6%	<0.001*
Corega vs. Listerine	85% ± 3%	87% ± 2%	0.331
Control vs. Toothbrush	85% ± 3%	64% ± 6%	<0.001*
Control vs. Listerine	85% ± 3%	87% ± 2%	0.168
Listerine vs. Toothbrush	87% ± 2%	64% ± 6%	<0.001*

**Table 4:** Wavelength-specific light transmittance (%T) values of polyethylene terephthalate glycol (PET-G) clear aligner specimens after 14 days of daily cleaning protocols.

Wavelength (nm)	Control (%)	Corega (%)	Listerine (%)	Toothbrush (%)
	Mean ± SD	Mean ± SD	Mean ± SD	Mean ± SD
350	71.32 ± 6.79	70.27 ± 7.21	69.54 ± 12.06	43.89 ± 8.69
400	81.67 ± 3.50	81.85 ± 2.89	83.57 ± 1.72	53.21 ± 8.21
450	83.11 ± 3.12	83.34 ± 2.84	85.09 ± 1.38	57.36 ± 7.53
500	84.13 ± 2.85	84.18 ± 2.89	86.09 ± 1.41	60.85 ± 6.91
550	84.90 ± 2.57	84.73 ± 3.11	86.78 ± 1.45	63.71 ± 6.32
600	85.40 ± 2.37	85.08 ± 3.25	87.28 ± 1.51	66.07 ± 5.78
650	86.01 ± 2.14	85.83 ± 3.45	87.99 ± 1.59	68.48 ± 5.39
700	86.80 ± 2.06	86.52 ± 3.90	88.77 ± 1.66	70.70 ± 4.93
750	87.54 ± 2.05	86.88 ± 4.06	89.25 ± 1.88	72.48 ± 4.57
800	88.14 ± 1.91	87.23 ± 4.32	90.08 ± 2.12	74.17 ± 4.18

Levene’s test indicated a violation of the homogeneity of variances assumption (P = 0.001). Therefore, Welch’s ANOVA was used, which revealed a statistically significant difference in light transmittance among the groups (P < 0.001). Post hoc pairwise comparisons using the Games-Howell test showed that the toothbrush group had significantly lower light transmittance compared to all other groups (P < 0.001). In contrast, no statistically significant differences were observed between the Control, Corega, and Listerine groups (P > 0.05).

To quantitatively compare the overall spectral performance, the area under the curve (AUC) was calculated for each specimen using the trapezoidal rule over the 350-800 nm range. The mean AUC values (arbitrary units) were the following: Control (38,045.9 ± 1,109.2), Corega (37,953.9 ± 1,478.0), Listerine (38,832.2 ± 801.4), and Toothbrush (28,650.6 ± 2,787.2). Welch’s ANOVA showed a significant difference among groups (P < 0.001). Post hoc Games-Howell tests revealed that the Toothbrush group had significantly lower AUC than each of the other three groups (P < 0.001 for all comparisons), while no significant differences were observed between the Control, Corega, and Listerine groups (P > 0.05).

Furthermore, to assess whether the differences between groups were consistent across the spectrum, separate Welch’s ANOVAs were performed at each measured wavelength (350-800 nm in 50-nm intervals). At every wavelength, the overall difference among groups was statistically significant (P < 0.001). Games-Howell post hoc tests confirmed that the Toothbrush group had significantly lower transmittance than the Control, Corega, and Listerine groups at all wavelengths (P < 0.001 for each pairwise comparison), whereas no significant differences were observed among the Control, Corega, and Listerine groups (P > 0.05).

## Discussion

The increasing use of clear aligner therapy reflects the high value patients place on near‑invisibility. Accordingly, optical integrity should be evaluated using objective, measurable parameters, such as light transmittance and/or spectrophotometric translucency, rather than subjective “clarity” alone. PET-G is commonly used in thermoformed aligners due to its non‑crystalline, amorphous copolyester structure, which provides excellent transparency and desirable optical properties. Indeed, PET-G aligners have been shown to exhibit high light transmittance within the visible spectrum compared with other commonly used aligner materials, with transparency related to crystallinity and microstructural organization [12]. However, thermoplastic dental polymers are not inert under clinical conditions, as staining agents and cleaning procedures can alter both surface characteristics and optical properties over clinically relevant periods. In PET-G systems, for example, mechanical brushing has been directly associated with changes in surface topography, which is a key factor influencing light scattering and, consequently, light transmittance [13].

In this context, the present findings demonstrate clear differences between mechanical and chemical cleaning approaches. Toothbrush-toothpaste cleaning resulted in the greatest reduction in light transmittance, whereas the control and effervescent-tablet groups remained close to baseline values, while mouthwash immersion produced a small increase in transmittance. The pronounced reduction in light transmittance observed in the toothbrush-toothpaste group may be explained by previous evidence indicating that mechanical brushing can alter surface topography and create microscopic irregularities capable of increasing light scattering [13]. Although such surface changes were not directly evaluated in the present study, the current findings are also consistent with previous long-term retainer studies reporting that repeated cleaning procedures, particularly mechanical toothbrushing, may reduce light transmittance over time [14]. By contrast, the relative stability observed in the effervescent tablet group is consistent with short-term studies in which such solutions are commonly used without consistently producing the greatest reductions in translucency across different materials [15]. Nevertheless, chemical cleaning cannot be assumed to be optically neutral. Previous studies have shown that outcomes depend on the balance between stain removal and preservation of surface integrity. For example, coffee staining has been reported to significantly reduce transmittance, while different cleaning methods produce distinct surface effects: mechanical cleaning tends to introduce linear abrasions, whereas chemical cleaning may leave residual surface deposits. Ultrasonic cleaning, in contrast, appears most effective in restoring transmittance without substantial surface damage [6]. These observations provide a coherent mechanistic perspective explaining why some chemical protocols may preserve optical clarity through efficient removal of deposits without excessive abrasion, while others may negatively affect it due to residue accumulation or polymer-solution interactions.

Although the Listerine group in the present study showed a slightly higher mean light transmittance than the control, this difference was not statistically significant and should be interpreted accordingly. The observed trend can be linked to the removal of superficial deposits, which can temporarily enhance apparent clarity. However, evidence from longer-term studies suggests that this effect may not persist. For instance, in a six-month investigation of copolyester retainers, the mouthwash (Listerine) group showed the largest decrease in transmittance, an effect attributed to ethanol and essential oils, which may affect material properties over time [16]. Similarly, a controlled three-month study reported that alcohol-based mouthwash reduced light transmittance in copolyester retainers [17]. Taken together, these findings support a time-dependent model for the effects of mouthwash: short-term exposure may temporarily improve apparent transmittance by removal of superficial deposits, whereas prolonged or repeated exposure (particularly to alcohol‑containing rinses) may promote sorption, plasticization, or microstructural changes that eventually impair optical properties.

Finally, some methodological considerations should be considered. A 15-minute daily immersion in Listerine was used based on a previously published protocol by Agarwal et al. [14], who examined the effects of mouth rinses on similar polymeric materials, thereby providing a practical reference for our design. This exposure regimen was chosen to represent an intensified daily chemical challenge, allowing measurable changes to be detected within a relatively short experimental period (14 days). However, it is important to note that this duration was selected for experimental standardization purposes and does not represent a direct clinical recommendation for patient use, as the effects of such an intensified regimen on other material properties were not evaluated in this study

Limitations of the current study

This in vitro study design cannot fully capture the complexity of the oral environment, where factors such as salivary enzymes, temperature fluctuations, dietary pigments, and mechanical forces may influence aligner optical properties over time. In addition, only one material type (PET-G) and a single brand were evaluated, which may limit the generalizability of the findings to other aligner materials with different compositions. Although standardized procedures were employed, manual toothbrushing may still introduce operator-dependent variability. Finally, although only a single measurement was recorded per specimen, the high reproducibility, as indicated by the ICC (ICC = 0.993), supports the reliability of the measurement protocol.

## Conclusions

Within the limitations of this in vitro study, mechanical cleaning with a toothbrush and toothpaste resulted in a noticeable reduction in light transmittance, indicating a negative impact on optical clarity. In contrast, chemical cleaning methods, including effervescent tablets and mouthwash, maintained light transmittance levels, with no significant differences from the control group. These findings suggest that non-abrasive cleaning approaches may be more suitable for preserving the optical properties of clear aligners. Further in vivo studies are recommended to confirm these outcomes under clinical conditions.
